# Immunochromatography Lateral Flow Strip Enhancement Based on Passive Gold Nanoparticles Conjugation to Detect *Schistosma haematobium* Antigens in Human Serum

**DOI:** 10.1007/s11686-024-00841-y

**Published:** 2024-05-16

**Authors:** Mahmoud N. El-Shall, Ibrahim Aly, Alaa Samen, Wesam M. Salama, Fadi Baakdah

**Affiliations:** 1 Department of Parasitology, Theodor Bilharz Research Institute, Giza, Egypt; 2 Zoology Department, Faculty of Science, Al-Azhar University, Cairo, Egypt; 3 Zoology Department, Faculty of Science, Tanta University, Tanta, Egypt; 4 Department of Medical Laboratory Science, Faculty of Applied Medical Sciences, King Abdulaziz University, 21589 Jeddah, Saudi Arabia; 5 Special Infectious Agents Unit, King Fahd Medical Research Center, King Abdulaziz University, 21589 Jeddah, Saudi Arabia

**Keywords:** *Schistosoma haematobium*, AuNPs, Diagnosis, Immunochromatography lateral, Flow strip (ICLFS), ELISA

## Abstract

**Purpose:**

This study aimed to develop and evaluate a lateral flow card for the detection of active *Schistosoma haematobium* infection.

**Methods:**

In order to prepare the immunochromatography lateral flow strip (ICLFS), antibodies purified from schistosomiasis were conjugated passively with gold nanoparticles using a potassium carbonate buffer.

**Results:**

The novel ICLFS was able to correctly identify 64 out of 67 samples of schistosomiasis, 6 out of 90 samples of other parasites, and 0 out of 27 control samples. Sensitivity, specificity, negative predictive value (NPV), and positive predictive value (PPV) were 95.5%, 93.3%, 90%, and 91.4% respectively. Comparatively, the sensitivity, specificity, NPV, and PPV of sandwich enzyme-linked immunosorbent assays (ELISA) conjugated with gold nanoparticles (AuNPs) were 91.1%, 88.8%, 85.9%, and 84.4% respectively. The increased sensitivity and specificity of ICLFS produced superior results to those of sandwich ELISA.

**Conclusion:**

In conclusion, ICLFS is more beneficial and precise than sandwich ELISA for detection of *S. haematobium* infection at early stage.

**Supplementary Information:**

The online version contains supplementary material available at 10.1007/s11686-024-00841-y.

## Introduction

Human schistosomiasis is a pathological illness caused by the parasitic infection of blood flukes classified under the genus *Schistosoma*. The World Health Organization (WHO) generally refers to this disorder as bilharzia [1]. Schistosomiasis is the most lethal and the second most prevalent parasitic disease in the world subsequent to malaria [2]. The highest incidence of schistosomiasis is observed in pre-school children, schoolchildren, and people whose occupations involve direct contact with infested water, particularly in poor countries and villages lacking a safe source of drinking water and adequate sanitation [3, 4]. WHO estimates that there are approximately 251.4 million people in 51 countries; at least 90% of those requiring treatment for schistosomiasis reside in Africa and still require preventive treatment following the Global Program for the elimination of Schistosomiasis; and at least 600 million are at risk of infection [4, 5]. The estimated annual mortality rate owing to schistosomiasis ranges in a periods of 2013 to 2016 from 200,000 to 24,072 [6, 7]. While, in 2023, the annual mortality rate due to schistosomiasis reached to 11,792 [1].

*Schistosoma haematobium* or the urogenital schistosomiasis is primarily prevalent in fifty-three countries, predominantly located in Africa and the Middle East, with overall prevalence rate of 1.3% [8]. The main factor contributing to the pathogenic nature of *S. haematobium* is the delay in the hypersensitive response, resulting in the formation of granulomas that encapsulate eggs. Subsequently, fibrosis occurs, leading to obstructive symptoms in the urinary system. The primary symptoms associated with *S. haematobium* infection primarily manifest as genitourinary diseases, including cystitis, dysuria accompanied by terminal hematuria, dull suprapubic discomfort, spermatorrhea, and hemospermia [9].

The success of worldwide programs aimed at eliminating schistosomiasis has been significantly influenced by the discovery of cost-effective and time-efficient diagnostic screening methods that exhibit high sensitivity. Over the past few decades, there has been significant progress in the development of various diagnostic procedures, ranging from basic microscopic detection to more advanced molecular assays [10]. Other screening methods involve the identification of antibodies; however, this approach has many limitations, particularly in regions with high prevalence rates. One major drawback is its inability to distinguish between different species of *Schistosoma* parasites or accurately detect and classify infections that are mild, acute, or chronic in nature. Moreover, the lack of precision in false-positive outcomes can be attributed to the phenomenon of cross-reactivity with other helminth diseases [11]. In terms of efficacy and utility, antigen detection emerges as the most advantageous and effective approach by enzyme-linked immunosorbent assays (ELISA), primarily owing to its remarkable sensitivity and capacity to discern between various stages of infection, encompassing both active and chronic states [12].

Nanotechnologies are increasingly recognized as potent tools that have the potential to overcome the limitations associated with conventional diagnostic techniques. The utilization of nanotechnology in immunoassays has significant promise in enhancing sensitivity, reducing the need for large sample volumes, and facilitating rapid and precise outcome generation. According to Padmavathy and Astha [13], the extensive surface area possessed by nanoparticles facilitates the binding of several molecules that are unique to the target of interest, hence enabling highly sensitive detection. Gold nanoparticles, commonly referred to as GNPs or AuNPs, have superior physical and chemical characteristics in comparison to their larger-scale counterparts. Due to their biocompatibility and stability, these substances are frequently employed in medical examinations for the purposes of treatment and diagnosis [14]. The potential obstacle to the integration of the sandwich ELISA into clinical diagnostic protocols is worth considering. Hence, the present study demonstrates our improvement of the immunochromatography lateral flow strip (ICLFS) with the specific purpose of detecting *S. haematobium* antigens within human serum samples.

## Materials and Methods

### Study Design and Ethics Statement

This study was carried out at the parasitology department of the Theodor Bilharz Research Institute (TBRI) in Giza, Egypt. Between April 2021 and December 2022, a total of 184 individuals were sampled from several regions across Egypt. The participants underwent comprehensive history-taking as well as clinical and parasitological examinations. Prior to their inclusion, all patients provided informed consent in accordance with the guidelines set forth by the Human Research Ethics Committee of the Theodor Bilharz Research Institute.

### Patient Sample Collection and Preservation

Urine and blood samples were collected from each participant: Urine samples underwent parasitological examination via sample sedimentation (10 ml of each urine sample), and the sediment was examined under light microscopy, Olympus CX23 Binocular Microscope (China) for *S. haematobium* eggs. Serum samples were collected from patients by extracting roughly 3 ml of venous blood into empty tubes. Following the process of coagulation, the sera samples underwent separation using centrifugation at a speed of 2000 rpm for a duration of 10 min. Subsequently, the separated samples were fractionated into small Eppendorf tubes and preserved at a temperature of −20 °C until it was utilized.

### Antigen Preparation and Purification

Adult *S. haematobium* worms were harvested from infected hamster and placed in a Petri dish and subjected to 3 washes using a phosphate buffered saline (PBS)(137 mM NaCl, 2.7 mM KCl, 10 mM Na_2_HPO_4_, and 1.8 mM KH_2_PO_4_)pH 7.4 to get rid of any contaminants, maintaining a temperature of 4 °C. Subsequently, the washed adult worms were subjected to homogenization at a speed of 6163×*g* for a duration of 10 min at a temperature of 4 °C by using a physically adjustable high-speed emulsifying homogenizer (PRO250,PRO Scientific Inc, USA). Following homogenization, the resulting mixture was diluted with 3 ml of PBS, pH 7.4. The homogenate was utilized as a crude antigen derived from adult worms and was stored at a temperature of −20 °C until it was ready for use [15]. Antigen purification involved the utilization of DEAE Sephadex A-50 (Cytiva 17-0180-02, USA) for ion exchange chromatography as well as a Sephadex G-200 HR (Pharmacia fine chemicals, USA) column for gel filtration chromatography [16].

### Rabbit Polyclonal Antibody Production and Purification

The process of antibody production involved the administration of a priming dose of 1 mg of *S. haematobium* purified antigen to a 3-month-old New Zealand white rabbit weighing approximately 1.5 kg. The immunization was conducted intramuscularly (i.m.) at four distinct sites. The purified antigen was administered in combination with 50 µl of aluminum hydroxide solution (13 mg/ml). Following a period of 15 days, the rabbit received a booster dosage consisting of 0.5 mg of the purified antigen combined with 50 µl of aluminum hydroxide. Later, three further booster doses were administered at weekly intervals [17]. Later, the rabbit blood was collected, and the serum was extracted. Polyclonal antibodies targeting *S. haematobium* (S-pAb) were purified from the serum using ammonium sulfate precipitation and caprylic acid precipitation techniques, as described by McKinney and Parkinson [18].

### Passive Conjugation of Gold Nanoparticles with S-pAb

The gold nanoparticles (AuNPs) with size average 20 ± 4 nm were purchased from a Nanotech Company for Photo-electronics (6th of October city, Giza, Egypt). Briefly, the pH of 20 mM AuNPs was adjusted to 8.0 using a 0.2 M K_3_CO_3_, S-pAb underwent dialysis against a 0.02 M sodium borate buffer (SBB) for a duration of 24 h at a temperature of 4 °C, with three successive changes of solution. A volume of 500 µl of S-pAb, which had been adjusted to the optimal concentration using a 50 µl of 10% NaCl solution as described in the study by Wang *et al*. [19], was introduced into a 2.5 ml solution of AuNPs at a pH of 8.0. The mixture was then vortexed to ensure proper suspension and then incubated in a shaker incubator for a duration of 20 to 40 min at a temperature of 37 °C with moderate agitation. Following that, 300 µl of 10% bovine serum albumin (BSA) in a 20 mM sodium borate solution was added to effectively block any remaining surface of AuNPs. The mixes were thereafter incubated for a duration of 20 min at room temperature (RT) prior to undergoing centrifugation at a speed of 9447*g* for a period of 45 min at a temperature of 4 °C. This centrifugation process was repeated three times. Following the last centrifugation step, the sedimented particles were reconstituted in a 2 ml solution of 0.02 M SBB containing 0.05% sodium azide, 2% BSA, 500 mM NaCl, 0.2% Tween 20, and 3% sucrose. Lastly, the AuNPs-rabbit anti-*S. haematobium* polyclonal antibody (Au–S-pAb) conjugates were stored at a temperature of −20 °C prior to their utilization [20]

### Fabrication of Au–S-pAb Lateral Flow Strips

The ICLFS of this work is composed of five main parts: a sample pad, a conjugation pad, an adjustment pad, a nitrocellulose flow membrane, an absorbent pad, and a semi rigid polyethylene adhesive sheet (SRP). The preparation of each part will be discussed in detail.

### Preparation of Sample and Conjugation Pads

**Fig. 1:**
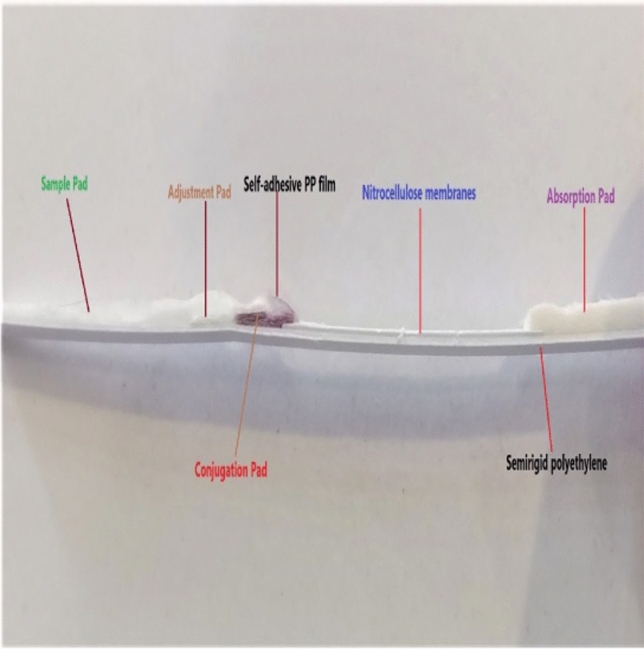
Fabrication of the lateral flow assay device

**Fig. 2:**
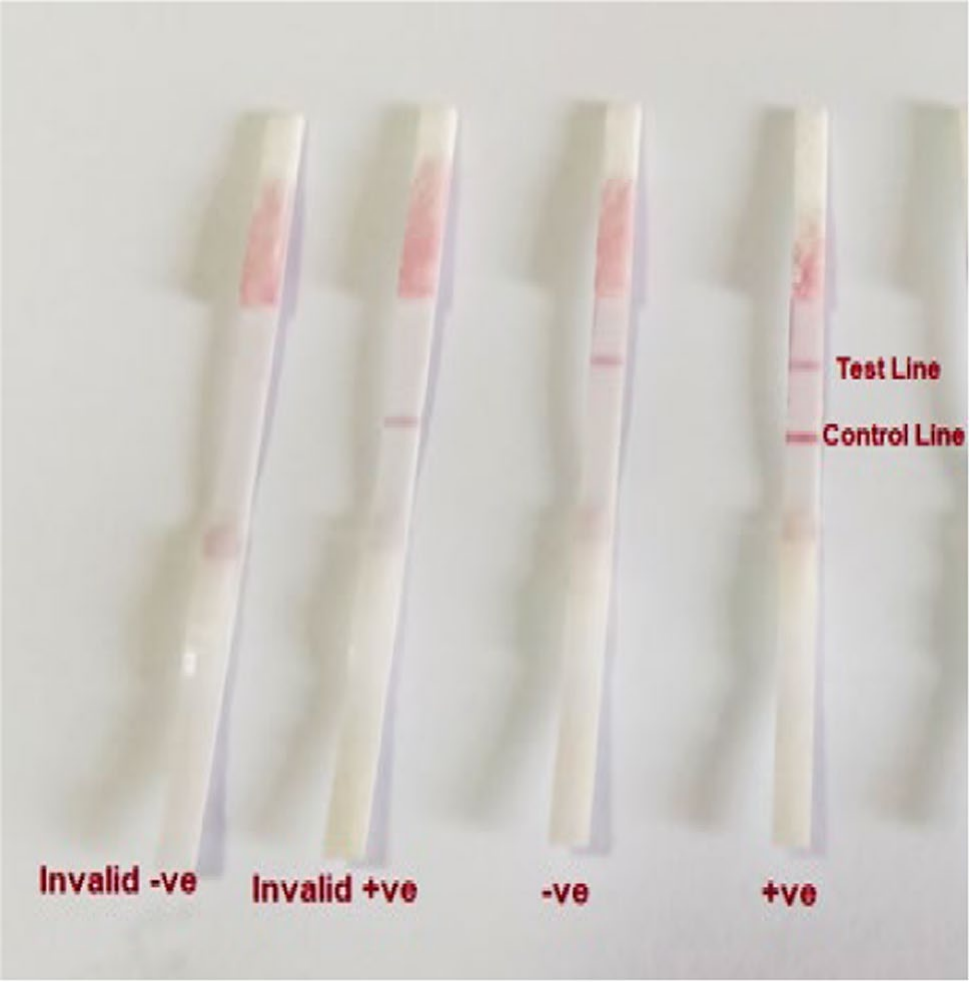
**A** Valid and invalid strip results, **B** Testing of human serum samples by ICLFS

Two glass fiber pads were used, specifically the LF1 type for sample pads and the standard 17 type for conjugation pads. Both pads were purchased from GE Healthcare Life Sciences. The pads underwent immersion in a treatment solution consisting of 0.01 mM PBS (pH 7.4) containing 0.2% Tween 20, 0.1% sodium azide, and 0.1 M NaCl for a duration of 1 min at RT (Fig. 1 supplementary)**(**all reagents from sigma-aldrich**)**. Subsequently, the pads were dried at a temperature of 45 °C for a period of 35 min using an air-drying oven (SI Series shaking incubator, China**)**. Then, 2 ml of the conjugate solution was sprayed onto the conjugation pad without any dilution using an Air-Jet sprayer with a spraying power of 15 μl/cm **(**Dispenser HPY001 from Werfen Equipment Co, China**)**(Fig. 2 supplementary). The pads were then dried again at 45 °C for 35 min using the air-drying oven. Finally, the pads were cut into dimensions of 1.3 × 30 cm^2^ and 0.5 × 30 cm^2^(sample and conjugation pads, respectively) by Using ECHO.01 cutter, ECHO for Medical Equipment Co, Egypt**.**

### Adjustment Pad Treatment and Preparation

This step involved immersing a VF2-type glass fiber pad in an adjustment solution. The solution used was 0.01 mM PBS (pH 7.4) containing 0.1 M NaCl, 0.2% Tween 20, 3% sucrose, and 0.05% sodium azide. The immersion process lasted for 2 min at RT. Subsequently, the pad was dried at 50 °C for 35 min using an air-drying oven. Finally, the dried pad was cut into pieces measuring 0.5 × 30 cm^2^.

### Preparing the Nitrocellulose Membrane Flow Strip Components Orientation

**Fig. 3:**
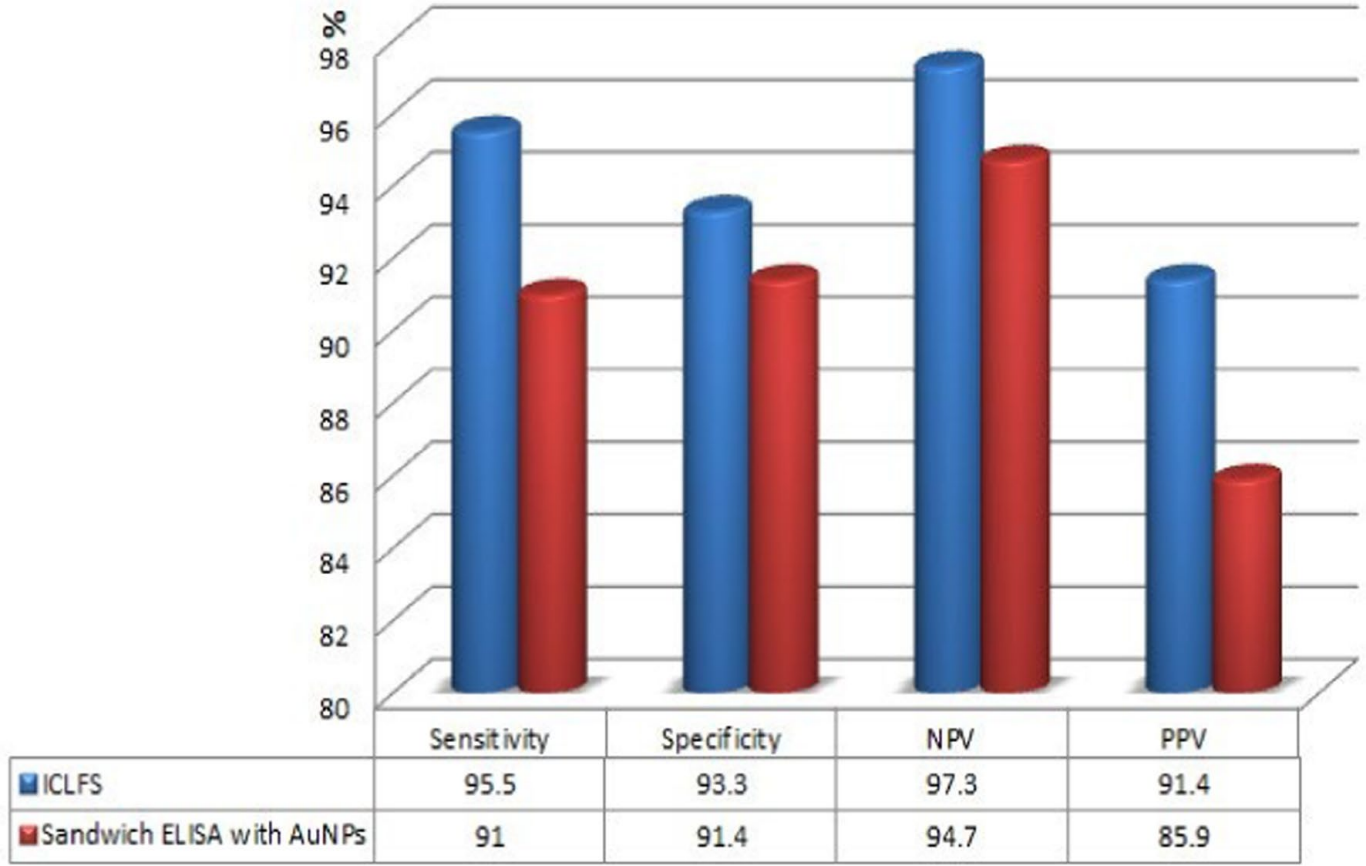
Histodiagram for assessment of sensitivity, specificity, NPV, and PPV using ICLFS and Sandwich ELISA

S-pAb and rabbit anti-human antibodies (Rαh-Ab) were diluted to a working concentration of 1 mg/ml in PBS (pH 7.4) to be installed on the test and control lines, respectively. The solutions were applied over the nitrocellulose membrane of the FF80HP type in a 1 μl volume/cm^2^. Maintaining a spacing of 0.5 cm between each line (Fig. 3 supplementary). Subsequently, the NCM was subjected to a drying process at 37 °C for 30 min using an air-drying oven. Then, the membrane was divided into strips of 2.5 × 30 cm^2^.

### Preparation of the Adhesive Sheet

The adhesive sheet (double-sided adhesive) was cut into segments measuring 6.5 × 30 cm^2^, serving as the backbone of the structure.

### Self-Adhesive Polyethylene Protective Transparent Film

A self-adhesive polyethylene film of low viscosity (PE)of 60-micron polyvinyl chloride (PVC) was cut into 1 × 30 cm^2^ segments and used as a protective layer for the conjugation pad and its overlapping site with NCM, and the adjustment pad and its overlapping site with the sample pad (Fig. 1).

### Assembly of ICLFS Components

The sandwich immunoassay served as the foundation for the ICLFS method. All assay components were assembled on SRP. Briefly, the NCM was fixed on the center of SRP (top side), and one pole of the NCM was followed by the conjugation pad with a 2 mm overlap at the NCM end. Moreover, the adjustment pad also has a 2 mm overlap with the conjugation pad end. Furthermore, the sample pad also overlaps with the adjustment pad end in a 2 mm space. On the other pole of the NCM, the absorption pad overlaps with it in a 2 mm space as well. It is important to note that the adjustment and conjugation pads were covered with a protective film that overlapped the sample pad and NCM by 2 mm. lastly, the ECHO1 cutter was used to slice the assembled master card into 2.7 mm test strips.

### Optimization and Testing of Human Serum Samples by ICLFS

Collected human serum samples of different volumes (5 µl, 10 µl, 15 µl, 20 µl, and 25 µl) were diluted in 100 µl PBS, added to sample pads, and allowed to laterally flow. After 10 min, the color development of the segments was examined. The presence of two-colored, test, and control lines, signifies a positive diagnosis. A single-colored control line devoid of a sample line indicates that the sample was negative. However, the test was rendered invalid if no colored lines appeared for the control or sample or if a colored line appeared only in the sample line but not the control (Fig. 1).

### Determine the Optimal pH Concentration of AuNPs and Protein

To determine and adjust pH concentration of AuNPs and protein, four different pH solutions [pH 7.5 solution of 4-(2-hydroxethyl)-1piperazineethanesuulfonic acid (HEPES), pH 8.5 PBS, pH 9.5 and10.5 solutions of potassium carbonate buffer (PCB)] were prepared. Thereafter, were added 20 µl of each pH solution then serial dilutions of proteins (5 µl, 10 µl, 15 µl and 20 µl), respectively. Next, vortex for 5 s and rotated the mixture for 10 min. Subsequently, 50 µl from each mixture was mixed separately with 50 µl of 10 NaCl solution (1:1 V:V), to verify the stability of AuNPs conjugates, vortexed for 5 s and the mixture was rotated for 1 h at room temperature. The determination of the optimum concentration of Absand pH were monitored by observing the changes in the mixture’s color.

### Nano-ELISA

ELISA Micro-titration plates were coated with 100 µl/well Au–S-pAb (5 μg/ml carbonate buffer, pH 9.6) and incubated overnight at RT. Plates were washed 3 times with 0.1 M PBS-tween20 (PBS/T), pH 7.4. The remaining sites were blocked by incubating 200 µl/well containing 2.5% BSA and PBS/T at 37 °C for 1 h. PBS/T was used 3 times to wash the plates. After determining the optimal concentration by standardizing the sandwich ELISA against *Schistosoma* antigens and antibodies, 100 µl of serum samples were pipetted at 37 °C for 1 h in duplicate wells. Following 3 washes, 100 µl/well of horseradish peroxidase-conjugated pAbs was added and incubated at RT for one hour. The plate was rinsed with a washing buffer 3 times. Subsequently, each well was filled with 100 µl of substrate solution (one tablet of OPD (Sigma) dissolved in 25 ml of 0.05 M phosphate citrate buffer, pH 5, with urea hydrogen peroxidase (Sigma), and the plate was incubated for 30 min in the dark at RT. Lastly, 50 µl of 8 N H_2_SO_4_ was added to each well to inhibit the enzyme–substrate reaction, and the absorbance was measured using an ELISA reader at 492 nm.

## Statistical Analysis

Data are presented as the mean ± standard deviation (Mean ± SD). The mean and SD values of each group were calculated from the mean values of all negative control results.The cutoff values were calculated by summation of the mean OD reading + 2 SD of negative controls. The mean groups were compared by analysis of variance. Calculations of sensitivity, specificity, agreement, PPV, and NPV were carried out as described by Hussein *et al*. [21].

## Results

### Participant's Sample Analysis

Not all 184 urine samples provided showed *S. haematobium* eggs post-urine sedimentation and microscopy analysis; only 67 out of 184 (36.4%) were *S. haematobium*-infected patients; group II: 90 out of 184 (48.9%) were from other parasite-infected patients; and 27 out of 184 (14.6%) were served as healthy control persons. Furthermore 48 out of 67 (71.6%) were mild *S. haematobium* infection; 12 out of 67 (17.9%) were moderate *S. haematobium* infection; and 7 out of 67 (10.4%) were high* S. haematobium* infection.

### Optimal pH Concentration of AuNPs and Protein

Many factors were optimized to improve the sensitivity and specificity of ICLFS, including the differences between the pH of AuNPs and proteins. The isoelectric point (pI) on the surface of AuNPs should be 0.5 pH units more or higher than the pH of proteins. Thus, the pI value of the AuNPs solution was adjusted slightly higher than the pI value of proteins prior to conjugation. Therefore, the optimal pH of AuNPs was pH 8, while the pH of the proteins was pH 7.4. Moreover, the optimal ratio of AuNP-IgG conjugate was determined to be 1:5 v/v. Lastly, the minimum amount of human serum required to develop color on the strip was 10 µl, compared with other concentrations of human serum that were tested.

### Detection of *S. haematobium* Antigens Using ICLFS

When ICLFS test strips were incubated with—*S. haematobium*-positive serum samples (n = 67), 64 out of the 67 positive samples gave a positive result with a strong red color in the test lines, which means that 3 *S. haematobium-*positive samples validated by light microscopy were not detected by ICLFS. The 3 false negative sera belonged to patients of mild infection group, while moderate and high infection groups reported positive results with all samples with a sensitivity of 95.5%. Moreover, in serum samples (n = 90) that had parasites other than *S. haematobium*, 4 had *F. gigantic*, and 2 had hydatid disease were weakly positive results with a faint red color in the test line (Fig. 2) with a specificity of 93.3%. Furthermore, the healthy control group (group III, n = 27) gave a negative result with all the group samples. The negative predictive value and positive predictive value were 97.3% and 91.4%, respectively (Fig. 3).

### Detection of Schistosomal Antigen Using Nano-sandwich ELISA

The mean optical density (OD) reading of negative controls and the standard deviation (SD) of the mean were computed to define the cut-off value for positivity or the line of demarcation between positive and negative results. The results of the tested samples with OD values greater than the cut-off value were recorded as positive for *S. haematobium* patients. Tested samples with OD values equal to or lower than the cut-off were recorded as negative for *S. haematobium*. The cut-off value for positivity was 0.232 when detecting schistosomiasis serum Ag by using AuNPs with sandwich ELISA. Herein, 61/67 serum samples from group I gave positive results, while 6 cases were negative (4 from group I and 2 from group II), with an OD of 0.68 ± 0.33 and a sensitivity of 91%. Moreover, 10 serum samples were positive in patients with other parasitic infections (group II), while the other 80 cases were negative, with an OD value of 0.3 ± 0.13. All healthy control patients were negative, with an OD value of 0.26 ± 0.03. The specificity was 91.4%, and the negative predictive value (NPV) and positive predictive value (PPV) were 94.7% and 85.9%, respectively (Fig. 3).

## Discussion

Urinary schistosomiasis is still a major health problem in endemic areas of Africa and the Middle East, affecting more than 110 million people in rural, agricultural, and peri-urban areas and people in contact with infested water. Individuals infected with *S. haematobium* are at risk of developing bladder cancer or renal failure later in their lives [22]. Suitable diagnostic tools for *S. haematobium* are becoming highly important for many reasons. For example, clinical diagnosis might lose its value because of a lack of specificity, and mass treatment might only remain cost-effective through the use of the appropriate economical diagnostic tools. In Egypt, *S. haematobium* is still causing serious health problems. Control programs over the last decade helped to decrease the prevalence of human schistosomiasis in Egypt; however, the disease is still endemic in many areas. The most reliable technique for epidemiological assessments of schistosomiasis is the microscopic examination for both intestinal and urinary schistosomiasis. This method provides a relatively easy-to-handle and inexpensive tool for detecting and estimating the concentration of *Schistosoma* eggs in fecal and urine samples [23]. However, the difficulties in meeting the multiple sampling requirements for classical parasitological diagnosis often lead to suboptimal results (because of the false-negative cases). Urine and blood samples are needed to be collected at specific times to recover the parasite eggs and antibodies, respectively [24]. Consequently, direct parasitological detection techniques are most often associated with poor sensitivity, which limits both the diagnosis of individuals with early or low-level infections and the evaluation of the efficacy of chemotherapy [25]. Antigen detection assays are the most useful alternative diagnostic tool due to their high sensitivity, allowing the diagnosis of active infection, and antigen assays allow direct measurement of worm burden compared to quantitative parasitological techniques that would be extremely valuable for immuno-epidemiological studies [26].The expression “assured” (“affordable, sensitive, specific, user-friendly, rapid and robust, equipment-free, deliverable to the end-user”) was used to describe the perfect or ideal diagnostic test or tool that is easily used in developing countries [27]. For monitoring and controlling *Schistosoma* infection in low-endemic areas, the most important characteristics of the diagnostic test are sensitivity and specificity. This study showed a highly increased sensitivity with a slight decrease in the specificity of ICLFS, which was caused by cross-reactivity with other nematodes like *F. gigantica* and occasionally with other serology tests like ELISA [28]. Many studies have indicated the applicability of the highly sensitive lateral flow test to diagnose patients with low levels of *Schistosoma* infection. In this study, we have developed ICLFS to detect *S. haematobium* antigens even if a patient has extremely low levels of *S. haematobium* antigens that cannot be detected by microscopic technique. Furthermore, this test is rapid (results are available in 10 min), very specific, sensitive, portable, and low-cost. In addition, unlike other tests that require blood samples at specific times during the day when the activity of parasites in the blood is high, this test may be done at any time of the day or night.

In the present study, ICLFS and sandwich ELISA were used to detect *S. haematobium* antigens. In cases of ICLFS group I, 67/184 (36.4%) were *S. haematobium*-infected patients, with 64 true positive cases out of 67. All negative results with *Schistosoma*-positive samples (n = 3) were produced from group I. Compared to sandwich-ELISA conjugated with AuNPs, there were 61 true positive cases out of 67; the negative results were detected in SgIandSgII, mild and chronic infections, respectively. The sensitivity for ICLFS and Sandwich-ELISA conjugated with AuNPs was 95.5% and 91%, respectively. In cases of individuals infected with other parasites as in group II (n = 90), ICLFS and Sandwich-ELISA with conjugated AuNPs detected 84 true negative cases out of 90 and 80 true negative cases out of 90, respectively. The cross-reactivity only occurred with *F. gigantica* and hydatid disease. However, cases of healthy control individuals in Group III (27/184, 14.6%) were negative inall the group samples. NPV and PPV for ICLFS and Sandwich-ELISA conjugated with AuNPs were 97.3%, 94.7%, 91.4%, and 85.9%, respectively.

## Conclusion

For screening and controlling the spread of NTDs, it is crucial to have a test that is user-friendly, quick, accurate, portable, and affordable. The use of ICLFS for the detection and estimation of *S. haematobium* antigens in blood samples from *S. haematobium*-infected individuals demonstrates a significant increase in sensitivity and specificity in comparison to the Nano-Sandwich-ELISA assay. This test is advantageous for patients because it decreases the time between diagnosis and treatment, thereby reducing the spread of infection without the need for a competent lab practitioner.

### Supplementary Information

Below is the link to the electronic supplementary material.

Figure 1 supplementary: Treatment of sample and conjugation pads in treatment solution for 1 min at RT. Figure 2 supplementary: Spraying of the conjugate solution onto the conjugation pad. Figure 3 supplementary: Dispensing the test and control line solutions onto the nitrocellulose membranes. Supplementary file1 (PPTX 557 KB).

## Data Availability

The datasets generated during and analyzed during the current study are available from the corresponding author on reasonable request.
