# Supplementary figures and images for: Immunochromatography Lateral Flow Strip Enhancement Based on Passive Gold Nanoparticles Conjugation to Detect Schistosma haematobium Antigens in Human Serum
Source: Acta Parasitol. 2024 May 16;69(2):1267–74. doi: 10.1007/s11686-024-00841-y (PMC11182813; doi:10.1007/s11686-024-00841-y)

## Slide 1
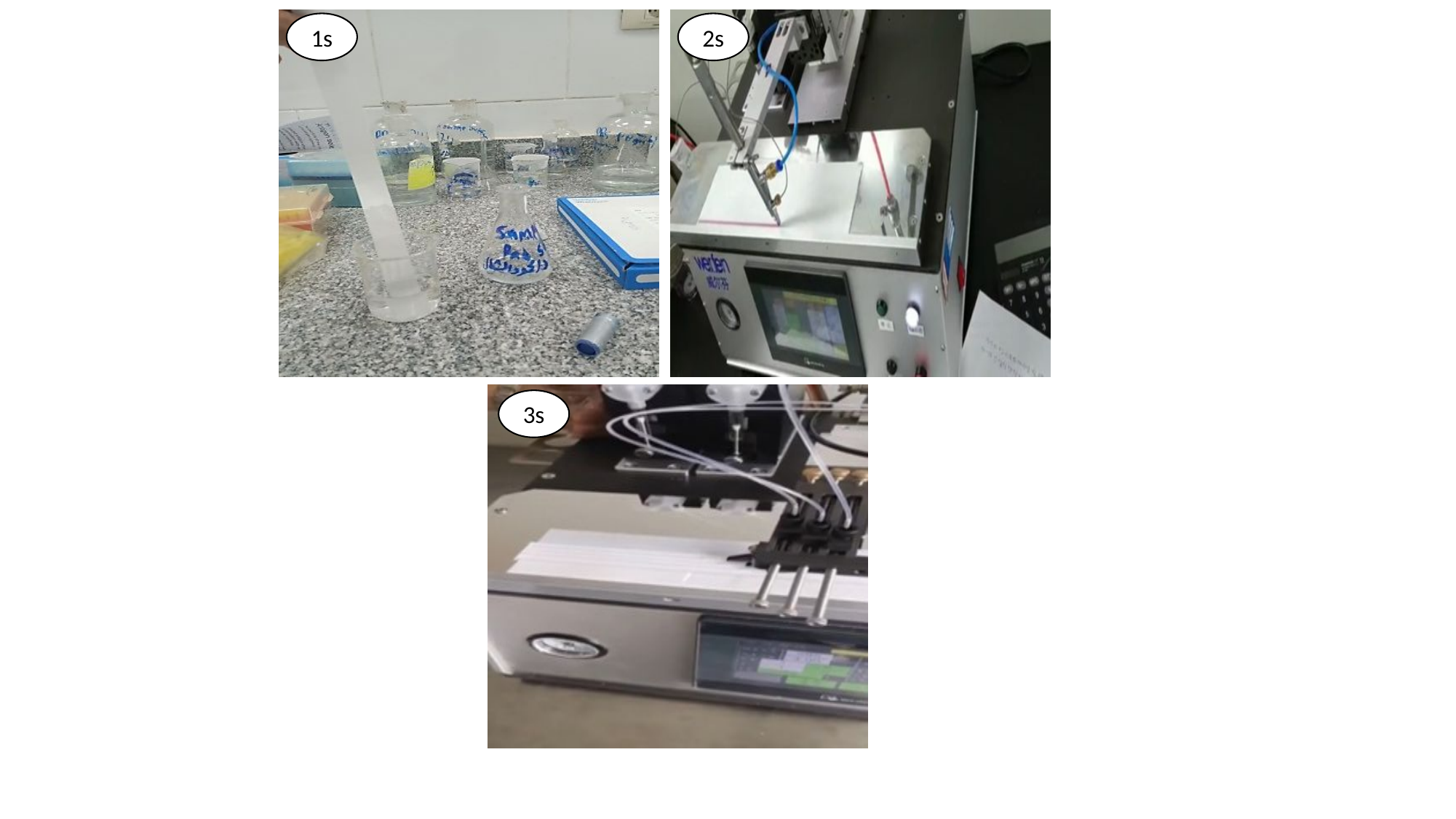

1s
2s
3s

Supplement: Supplementary file 1 — Figure 1 supplementary: Treatment of sample and conjugation pads in treatment solution for 1 min at RT. Figure 2 supplementary: Spraying of the conjugate solution onto the conjugation pad. Figure 3 supplementary: Dispensing the test and control line solutions onto the nitrocellulose membranes. Supplementary file1 (PPTX 557 KB). [file 11686_2024_841_MOESM1_ESM.pptx]
